# TMS Motor Thresholds Correlate With TDCS Electric Field Strengths in Hand Motor Area

**DOI:** 10.3389/fnins.2018.00426

**Published:** 2018-06-25

**Authors:** Marko Mikkonen, Ilkka Laakso, Motofumi Sumiya, Soichiro Koyama, Akimasa Hirata, Satoshi Tanaka

**Affiliations:** ^1^Department of Electrical Engineering and Automation, Aalto University, Espoo, Finland; ^2^Department of System Neuroscience, National Institute for Physiological Sciences, Okazaki, Japan; ^3^School of Health Sciences, Faculty of Rehabilitation, Fujita Health University, Toyoake, Japan; ^4^Department of Computer Science and Engineering, Nagoya Institute of Technology, Nagoya, Japan; ^5^Laboratory of Psychology, Hamamatsu University School of Medicine, Hamamatsu, Japan

**Keywords:** tDCS, TMS, resting motor threshold, electric field estimation, FEM

## Abstract

Transcranial direct current stimulation (TDCS) modulates cortical activity and influences motor and cognitive functions in both healthy and clinical populations. However, there is large inter-individual variability in the responses to TDCS. Computational studies have suggested that inter-individual differences in cranial and brain anatomy may contribute to this variability via creating varying electric fields in the brain. This implies that the electric fields or their strength and orientation should be considered and incorporated when selecting the TDCS dose. Unfortunately, electric field modeling is difficult to perform; thus, a more-robust and practical method of estimating the strength of TDCS electric fields for experimental use is required. As recent studies have revealed a relationship between the sensitivity to TMS and motor cortical TDCS after-effects, the aim of the present study was to investigate whether the resting motor threshold (RMT), a simple measure of transcranial magnetic stimulation (TMS) sensitivity, would be useful for estimating TDCS electric field strengths in the hand area of primary motor cortex (M1). To achieve this, we measured the RMT in 28 subjects. We also obtained magnetic resonance images from each subject to build individual three-dimensional anatomic models, which were used in solving the TDCS and TMS electric fields using the finite element method (FEM). Then, we calculated the correlation between the measured RMT and the modeled TDCS electric fields. We found that the RMT correlated with the TDCS electric fields in hand M1 (*R*^2^ = 0.58), but no obvious correlations were identified in regions outside M1. The found correlation was mainly due to a correlation between the TDCS and TMS electric fields, both of which were affected by individual's anatomic features. In conclusion, the RMT could provide a useful tool for estimating cortical electric fields for motor cortical TDCS.

## Introduction

Transcranial direct current stimulation (TDCS) is a non-invasive method of stimulating the brain and is capable of eliciting changes in cortical activity that outlast the stimulation period (Priori et al., 1998; Nitsche and Paulus, 2000, 2001). Studies suggest that these TDCS-induced changes have the potential to serve as a treatment for various cerebrovascular, psychiatric, and neurological diseases such as stroke (Marquez et al., 2015), depression (Meron et al., 2015), and schizophrenia (Fröhlich et al., 2016). However, this potential is hindered by inter-individual variations in its efficacy (López-Alonso et al., 2014; Wiethoff et al., 2014; Chew et al., 2015; López-Alonso et al., 2015) which may be related to differences in the induced electric fields (EFs).

Computational studies have suggested that these differences in the induced EFs may arise from anatomical differences between individuals: The distance from the surface of the scalp to the surface of the brain, in terms of subcutaneous fat thickness (Truong et al., 2013), skull thickness (Opitz et al., 2015), and especially the amount of CSF (Laakso et al., 2015; Opitz et al., 2015), has been found to have an effect on the electric fields in the adult brain. Similar results have also been found in children (Kessler et al., 2013; Fiocchi et al., 2016). In fact, induced cortical EFs may be a more useful parameter for determining the appropriate TDCS dose (Bestmann and Ward, 2017), compared to the input current that is commonly employed (Horvath et al., 2015) in TDCS studies.

As it is virtually impossible to non-invasively measure the strength of TDCS-induced EFs *in vivo*, the EFs are often modeled computationally. Unfortunately, estimating the EFs that are induced in a subject's brain with a computer model is a tedious process involving magnetic resonance imaging (MRI), segmentation of the acquired images, and computer simulations, making it an impractical approach in the clinical environment. Developing a simpler and more-robust method of estimating the strength of TDCS EFs would be beneficial because it would permit obtaining more-uniform TDCS stimulation intensities in terms of the induced EFs.

Transcranial magnetic stimulation (TMS) is a commonly used method for studying the excitability of the motor cortex (Ilmoniemi et al., 1999). TMS works by different mechanism from TDCS, magnetically inducing a brief pulsed EF that activates cortical neurons, which, in the case of motor cortical TMS, evokes responses that can be easily measured using electromyography. Theoretically, the EFs induced by TMS depend mainly on the distance below the scalp surface (Tofts, 1990). This has been confirmed in electrophysiological studies, which have shown that the scalp–cortex distance explains 50–70% of inter-subject variability in the motor threshold (MT) (Kozel et al., 2000; Stokes et al., 2007; Herbsman et al., 2009). Modeling studies have shown that, in addition to the scalp–cortex distance, the EFs induced by TMS are affected by the distribution of the CSF and orientation of the gyri with respect to the direction of the induced EF (Opitz et al., 2013, 2014; Laakso et al., 2014; Bungert et al., 2016; Laakso et al., 2018). These anatomical features, namely the thicknesses of the scalp tissues, skull, and CSF, as well as the orientation of gyri and sulci, also affect the EFs produced by TDCS (Datta et al., 2009; Truong et al., 2013; Laakso et al., 2015; Opitz et al., 2015). Therefore, the EFs of TMS and TDCS may be linked, despite the fact that TDCS and TMS act via different mechanisms. Based on this, our hypothesis was that the MTs measured using TMS may be indirectly related to the TDCS EFs.

Recent studies have indicated that individual TMS thresholds may indeed affect the after-effects of TDCS (Labruna et al., 2016; Jamil et al., 2017): Labruna et al. (2016) studied the relationship between TDCS efficacy and individual sensitivity to TMS using 1 mA anodal and cathodal stimulation. This was extended to a range of 0.5–2 mA by Jamil et al. (2017). Both studies found TMS thresholds to have a modest effect on the after-effects of anodal 1 mA TDCS at early epoch (0–30 min after stimulation). However, neither study found significant effects for cathodal stimulation, at later epochs, or for other stimulation currents.

The aim of the present study was to study whether TMS motor thresholds, namely the resting motor threshold (RMT), would also be a useful parameter for estimating the strength of TDCS EFs in the hand area of primary motor cortex (M1). We also investigated the relationship between the EFs of TDCS and TMS.

## Methods

### Subjects

Twenty-eight healthy subjects (7 women and 21 men; mean age ± standard deviation [SD] = 27.1 ± 6.4 years) participated in the study. All subjects participated in both the RMT measurements and the MRI. The subjects were neurologically healthy and had no family history of epilepsy. The Human Research Ethics Committee at the National Institute for Physiological Sciences approved all experimental procedures. All subjects provided both informed and written consent before participating in the experiment. Both the left- and right-handed subjects were included in this study, as no significant interhemispheric differences have been found in responses to TMS (Bashir et al., 2013).

### RMT measurement

We determined the RMT for the left abductor pollicis brevis muscle as a measure of cortical excitability using a figure-eight-shaped coil (diameter of the individual loop: 9 cm) connected to a Magstim 200 magnetic stimulator (Magstim Company, UK). The coil and stimulator were applied to elicit motor-evoked potentials (MEPs) in two separate sessions that were performed on different days. The coil handle was held perpendicular to the central sulcus. For each subject, the location of the hand M1 region (hand knob) was identified using an individual T1-weighted MR image and a frameless stereotaxic navigation system (Brainsight 2; Rogue Research, Montreal, Canada). For the RMT measurements, the coil was placed directly above the center of the hand knob, as identified by the navigation system. The RMT was defined as the lowest stimulation intensity required to elicit MEPs with a peak-to-peak amplitude of 50-μV in five of ten trials (Rossini et al., 1999).

### MRI

All MRI scans were acquired using a 3.0 T MRI scanner (Verio; Siemens, Ltd., Erlangen, Germany). Structural T1-weighted MRI of all subjects were acquired using a Magnetization Prepared Rapid Acquisition in Gradient Echo (MPRAGE) sequence (TR/TE/TI/FA/FOV/voxel size/number of slices = 1,800 ms/1.98 ms/800 ms/9° /256 mm/1.0 mm x 1.0 mm x 1.0 mm/176). In addition, T2-weighted MRI were acquired for the same subjects (TR/TE/FOV/voxel size/slice number = 4,500 ms/368 ms/256 mm/1.0 mm x 1.0 mm x 1.0 mm/224 slices).

### Volume conductor models

The MR-images were segmented with an in-house software (Laakso et al., 2015). Details of the segmentation process have been described previously (Laakso et al., 2015, 2016). In short, the FreeSurfer image analysis software (Dale et al., 1999; Fischl et al., 1999; Fischl and Dale, 2000; Desikan et al., 2006) was used for segmenting the brain. Non-brain tissues were segmented using a semi-automatic procedure that uses both T1 and T2 weighted MR images, which were first divided into three compartments: the scalp, skull and the contents of the skull (without brain). These compartments were then further segmented into individual tissues (see Table 1). The segmentation process also ensured that the minimum distance between the brain and the inner skull surface was not shorter than 0.5 mm. Volume conductor models with a resolution of 0.5 mm were built for each subject from the segmented data by assigning conductivity values to each voxel in a cubical grid. The tissue conductivities we used were assumed to be linear and isotropic.

**Table 1:** List of segmented tissues and the electric conductivities used in modeling TDCS and TMS.

**Tissue**	**σ_TDCS_ (S/m)**	**σ_TMS_ (S/m)**
GM	0.20	0.215
WM	0.14	0.142
CSF	1.8, (Baumann et al., 1997)	1.8, (Baumann et al., 1997)
Compact bone	0.008, (Akhtari et al., 2002)	0.009, (Akhtari et al., 2002)
Spongy bone	0.027, (Akhtari et al., 2002)	0.034 (Akhtari et al., 2002)
Fat	0.08, (Gabriel et al., 2009)	0.15, (Wake et al., 2016)
Skin	0.08, (Gabriel et al., 2009)	0.43, (Wake et al., 2016)
Muscle	0.16, (Gabriel et al., 2009)	0.18, (Gabriel et al., 2009)
Dura	0.16	0.18
Blood	0.7, (Gabriel C. et al., 1996)	0.7, (Gabriel S. et al., 1996)
Eye humor	1.5, (Lindenblatt and Silny, 2001)	1.6, (Lindenblatt and Silny, 2001)

For modeling TDCS, a gray matter conductivity of 0.2 S/m was selected, as existing literature suggests that its value typically varies from 0.1 to 0.3 S/m (Freygang and Landau, 1955; Stoy et al., 1982; Ranck, 1963; Gabriel C. et al., 1996; Latikka et al., 2001; Akhtari et al., 2006). Similarly, white matter conductivity is approximately 30% less than that of gray matter (Freygang and Landau, 1955; Stoy et al., 1982; Gabriel C. et al., 1996); thus, we used a white matter conductivity of 0.14 S/m. For modeling TMS, the gray and white matter conductivity values were extrapolated to the frequency of 3 kHz of the magnetic stimulator (Nieminen et al., 2015) using a Cole–Cole parametric model (Gabriel S. et al., 1996) from human *in vivo* values of 0.26 and 0.17 S/m measured at 50 kHz (Koessler et al., 2017), respectively. Thus, 0.215 S/m was used for gray matter and 0.142 S/m for white matter. The conductivity values for other tissues are presented in Table 1. The conductivity values for compact and spongy bone were increased by 30% to compensate for the room temperature measurements, and the dura conductivity was chosen arbitrarily to be the same as that of muscle.

### EF modeling

An in-house finite element method (FEM) solver (Laakso and Hirata, 2012), which employed the volume conductor model voxels as elements, linear basis functions, and the geometric multigrid method, was used to establish the electric scalar potential ϕ that was induced at the vertices of each voxel by TDCS and TMS stimulation.

**Figure 1:**
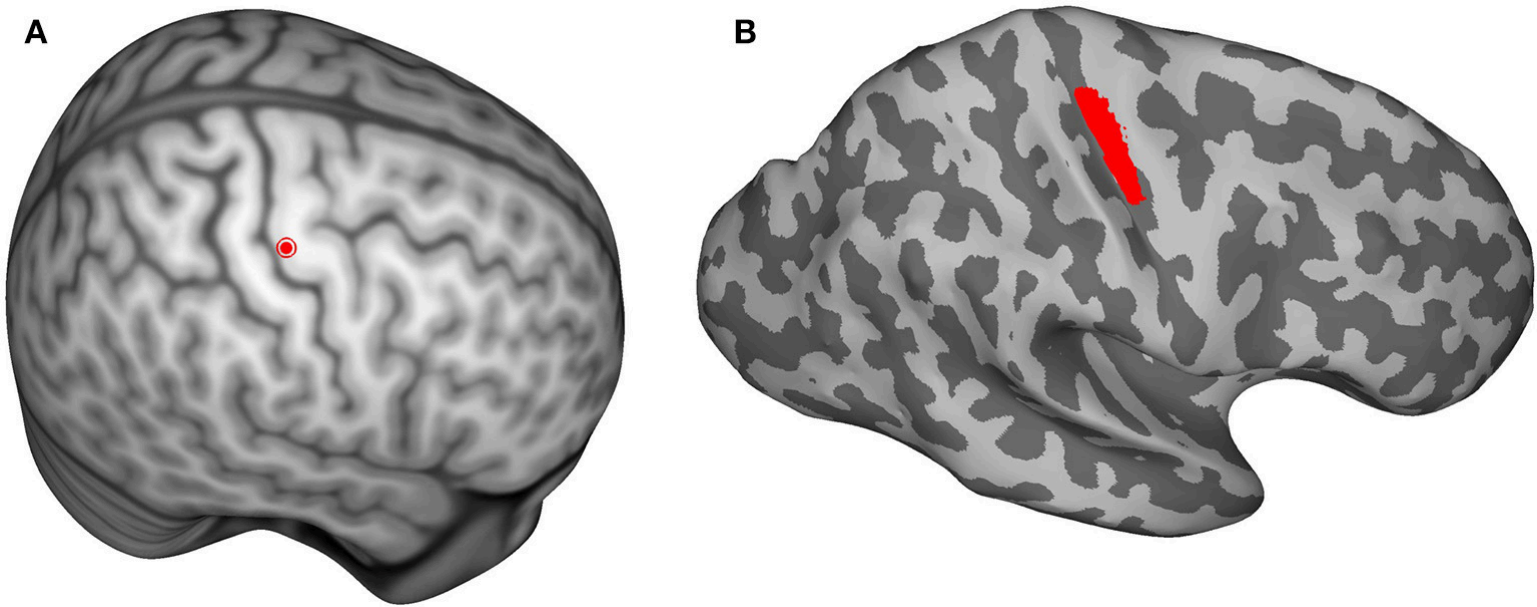
**(A)** The center of the hand knob is shown as a red dot on the Montreal Neurological Institute (MNI) template brain. The hand-knob location was mapped from the MNI template to each individual subject's brain using an inter-subject registration method in order to keep the anode and coil locations consistent. **(B)** The location of the region of interest (shown in red) that was used during data analysis. The shading represents the gyral structure (dark = sulci, light = gyri).

For TDCS, the solver was used to iteratively calculate ϕ from the potential equation

(1) $$\begin{array}{l} \nabla \cdot \sigma_{\text{TDCS}}\nabla \phi =0, \end{array}$$

where σ_TDCS_ is the electric conductivity. The iteration was continued until the relative residual of the numerical solution was less than 10^−6^, which typically results in less than 0.1% error in the EF (Laakso and Hirata, 2012). The EF was determined from $\vec{E}=-\nabla \phi$. The active electrode was located above the hand knob (Figure 1A) and the reference electrode was located at the contralateral forehead (Fp1) for each subject. The electrodes were modeled based on a realistic two-compartment design (Saturnino et al., 2015) consisting of 5 × 5-cm and 6-mm thick saline-soaked sponges (σ = 1.6 S/m) and a 1-mm thick rubber sheet (σ = 0.1 S/m). The connector was modeled as a disk with a radius of 5 mm that was located beneath the rubber sheet, with the current source/sink placed uniformly on the disk. The rubber sheet surrounded the connector, with 1 mm of rubber on all sides. A 1-mA input current was used.

To model TMS, the quasistatic approximation was used, i.e., the electric and magnetic fields were assumed to vary very slowly with time. This assumption is valid because the energy of the stimulation waveform is concentrated at frequencies lower than 10 kHz (Wang and Eisenberg, 1994). Furthermore, the magnetic skin effect can be ignored because the conductivities of biological tissues are very small compared to those of metals. Under these assumptions, the EF can be represented as $\vec{E}=-\nabla \phi -\frac{\partial }{\partial t}\vec{A}$, where *t* denotes time, and $\vec{A}$ is the magnetic vector potential. The scalar potential ϕ was determined with the following equation:

(2) $$\begin{array}{l} \nabla \cdot \sigma_{\text{TMS}}\nabla \phi =\nabla \cdot \sigma_{\text{TMS}}\frac{\partial }{\partial t}\vec{A}. \end{array}$$

Under the quasistatic approximation, the current in the coil windings was constant, and $\vec{A}$ was solved analytically via the Biot–Savart law using a coil current (1.74 A/μs, Laakso et al., 2018) that produced the same peak EF as a monophasic pulse of the Magstim 200 stimulator at 1% of the maximum output. The choice of the stimulator intensity for computer modeling is arbitrary as the induced EFs change linearly with stimulator output (Nieminen et al., 2015), and thus, EFs at any other stimulator output can be obtained by multiplication. The model of the figure-8 coil consisted of two circular wings of thin wire with nine windings each (Laakso et al., 2018). The outer and inner diameters of the wings were 9.7 and 7.2 cm, respectively. The dimensions were based on the Magstim 70-mm figure-8 coil (Thielscher and Kammer, 2004). The coil windings were located on a tangential plane above the hand knob at a height of 5.5 mm from the skin, to account for the thickness of the coil (1.1 cm), and oriented 45° from the anteroposterior direction.

In order to keep the anode and the coil locations consistent for each subject, the center of the hand knob was selected on the Montreal Neurological Institute (MNI) template brain (Figure 1A) and mapped to each subject's brain using an inter-subject registration method (Laakso et al., 2016). The closest point on the scalp to the mapped hand-knob center was where the center of the anode and TMS coil were positioned.

All simulations presented in this study were executed with MATLAB (version 2014a, Mathworks Inc., Natick, MA, USA) on a computer with 8-core Intel Xeon processor (3.4 GHz) and 32 GB of memory. On average, the models contained 33 million elements and took 45 s to solve.

### Data analysis

All data analyses were performed using MATLAB (version 2014a, Mathworks Inc., Natick, MA, USA). Linear regression analysis was used to study the correlation between RMTs of the two sessions. The absolute values of the EFs in each subject were determined on a surface located 1 mm below the gray-matter surface. The surface EFs were mapped onto the surface of the MNI template brain (Figure 1B) using a previously described inter-subject registration procedure (Laakso et al., 2016). The surfaces used in analyses are triangular meshes constructed using Freesurfer.

The region of interest (ROI; Figure 1B) was defined as the area on the MNI template brain surface within a probabilistic cytoarchitectonic map of Brodmann area 4, as defined by FreeSurfer (Fischl et al., 2007), that was within 1.5 cm of the center of the hand knob [MNI coordinates (Maki et al., 2008): x = 37.41, y = −24.00, z = 57.41]. The spatial mean and maximum EFs were calculated in the ROI for each subject. Linear regression analysis was used to examine the correlations between the RMTs and the TDCS EFs in the ROI, as well as the correlations between the TDCS EFs and TMS EFs in the ROI. Studentized residuals were used to find outliers in the analyses with 95% confidence interval, and the found outliers were omitted.

To study the spatial extent of the correlations, linear regression analyses between the RMTs and TDCS EFs as well as between the TDCS EFs and TMS EFs were performed nodewise on the triangular MNI brain surface mesh (consisting of 149,319 nodes). To exclude the nodes with low average TDCS EFs from the analysis, the analyses were only performed at the 31039 nodes where the subject-wise mean TDCS EF magnitude was higher than 50% of the maximum. The Benjamini-Hochberg procedure was used to control the false discovery rate (FDR) at a level of 5%.

## Results

### RMTs

Table 2 presents the subject handedness and measured RMTs. The level of RMTs remained consistent intra-individually between the two measurements, differing by only three percentage points on average and strongly correlating (*R*^2^ = 0.88, *P* < 0.0001). In contrast, the inter-individual variance was large, with the largest individual mean RMT (71%) being 40 percentage points higher than the lowest mean RMT (31%); for all subjects and both sessions, the mean and SD of the RMTs were 47.6% and 9.9%, respectively.

**Table 2:** Subjects' handedness and measured RMTs from both sessions.

**Subject**	**Handedness**	**RMT_*A*_ (%)**	**RMT_*B*_ (%)**
1	R	55	52
2	R	50	48
3	R	36	32
4	R	46	48
5	L	60	63
6	R	50	50
7	R	40	45
8	R	54	52
9	R	46	48
10	R	52	42
11	R	42	48
12	R	45	46
13	R	60	62
14	L	45	48
15	R	43	46
16	R	46	54
17	R	70	72
18	R	60	62
19	R	44	48
20	R	44	46
21	R	42	43
22	R	32	32
23	R	34	28
24	R	36	34
25	R	64	70
26	R	48	50
27	R	38	36
28	R	40	40

### EF modeling

**Figure 2:**
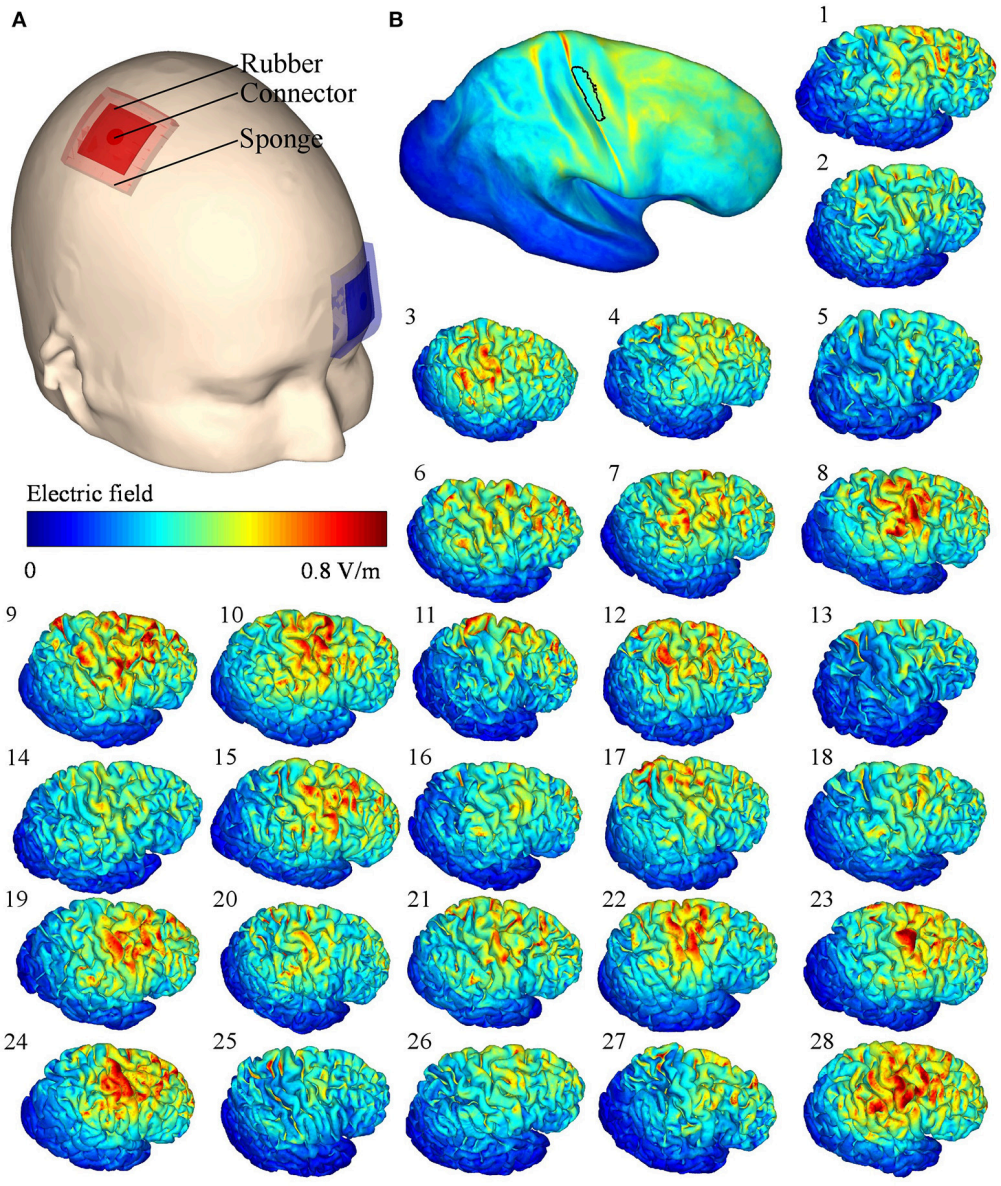
**(A)** Transcranial direct current stimulation (TDCS) electrode locations on the scalp. **(B)** Mean of the electric fields (EFs) mapped onto the Montreal Neurological Institute template, the black outlined area represents the region of interest; (1–28) simulated TDCS EFs of each subject on the right hemisphere.

The TDCS EFs in the right hemisphere for each subject are presented in Figure 2. Although the stimulation parameters were identical, the modeled EFs varied inter-individually. For all subjects, the mean ± SD of the maximum absolute EF in the ROI was 0.61 ± 0.09 V/m, with the highest and lowest maximum values being 0.85 and 0.47 V/m, respectively. The mean EF in the ROI was 0.34 ± 0.07 V/m.

### Correlation between the RMTs and TDCS EFs

**Figure 3:**
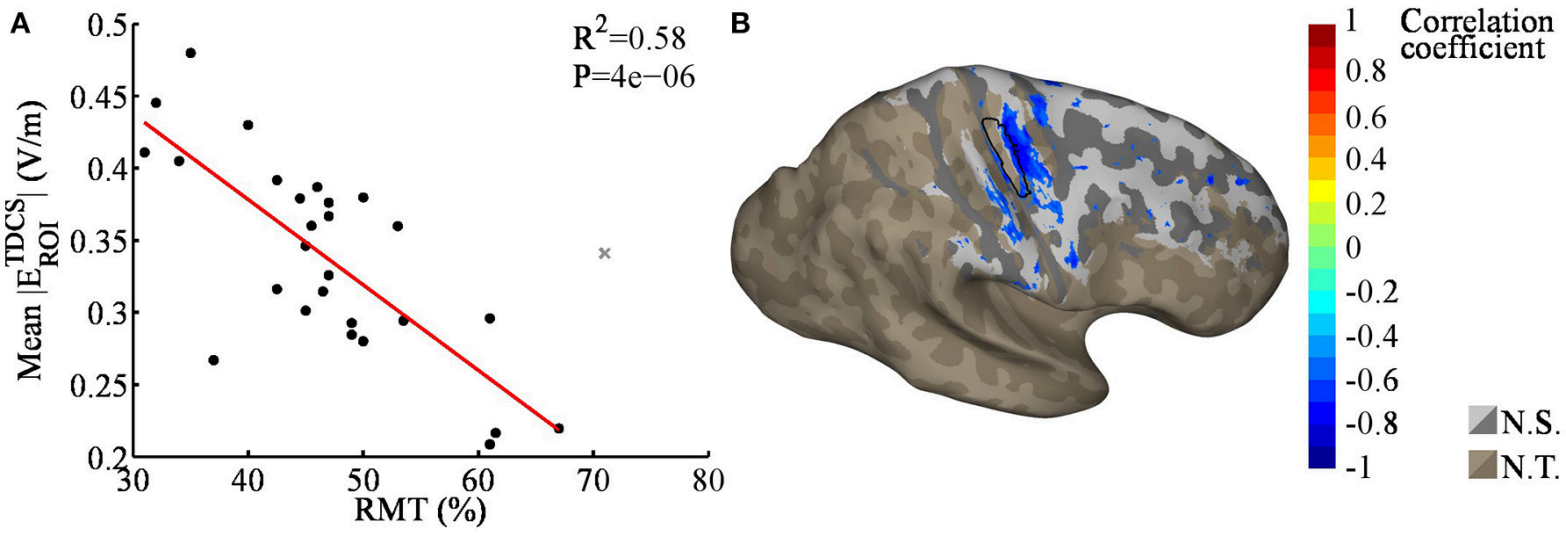
**(A)** Regression plot of the mean transcranial direct current stimulation electric fields (TDCS EFs) within the region of interest [ROI; black outlined area in **(B)**], as a function of the resting motor threshold (RMT); the gray cross marks the outlier that was omitted from the analysis. The RMT was found to correlate significantly with the TDCS EFs. **(B)** Nodewise correlation between the RMT and individual TDCS EFs; the significant correlation coefficients are shown in color, the non-significant coefficients in gray (N.S.), and the unstudied areas (average TDCS EF lower than 50% of the maximum) in brown (N.T.). The shading in the gray/brown areas represents the gyral structure of the brain (dark = sulci, light = gyri).

As the RMTs correlated significantly between sessions, we calculated the mean of the two RMT measurements for each subject and used the means in the analysis. Linear regression analysis revealed a significant correlation between the mean TDCS EF strengths and RMTs (*R*^2^ = 0.58, *P* < 0.001, see Figure 3A, regression coefficients are presented in Table 3), one data point was omitted based on the outlier analysis. Specifically, subjects with a higher RMT tended to have a smaller mean TDCS EF. Our nodewise examination (see Figure 3B) of the correlation between the TDCS EFs and RMTs revealed an area with a significant (with a 5% FDR) negative correlation beneath the TDCS electrode. This suggests that the EFs within this region could be estimated using the RMT. However, as seen in Figure 3B, the correlation between the RMT and the EFs in regions anterior to the precentral gyrus were not significant.

**Table 3:** Coefficients for linear regression EF = *E*_0_ + *k*×RMT + ϵ, presented in Figure 3A.

	**Predicted value**	**95% Confidence interval**
*E*_0_	0.6152	[0.5167, 0.7137]
*k*	−0.0059	[−0.0080, −0.0039]

*EF is the mean TDCS EF in the ROI (V/m), E_0_ is the intercept (V/m), k is the slope of the regression line (V/m per % of stimulator output), and ϵ is the residual*.

### Correlation between TMS and TDCS EFs

**Figure 4:**
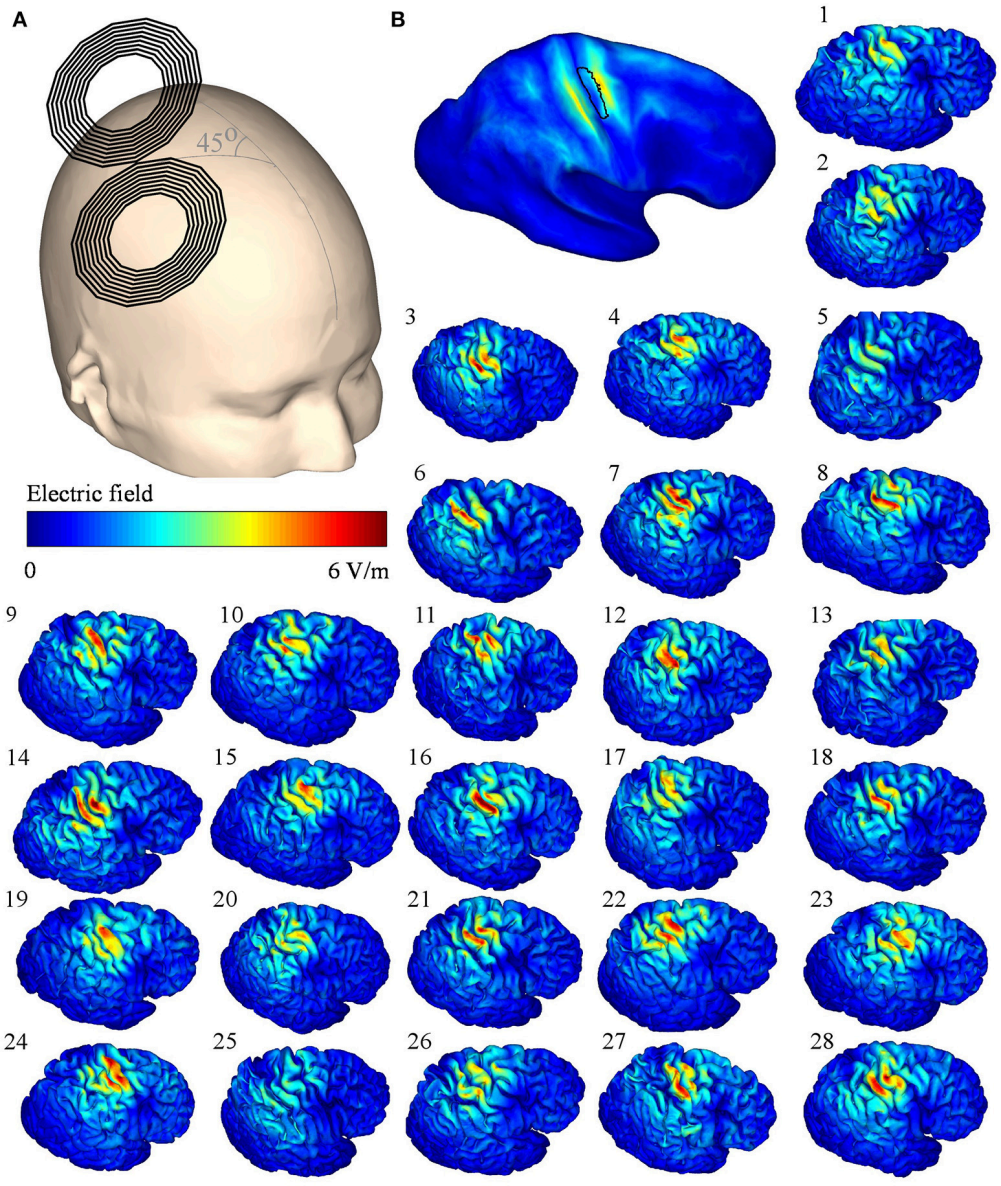
**(A)** Transcranial magnetic stimulation (TMS) coil orientation on the template head. **(B)** Mean of the electric fields (EFs) mapped onto the Montreal Neurological Institute template, the black outlined area represents the region of interest; (1–28) simulated TMS EFs of each subject on the right hemisphere. The TMS stimulator output was set to 1% of the maximum stimulator output.

As RMT is measured using TMS, we hypothesized that the EFs induced by TMS would be connected to those produced by TDCS, which could explain the correlation between the TDCS EF strengths and the RMT. To test this, we modeled the TMS-induced EFs in each subject. The modeled TMS EFs and their mean are presented in Figure 4. Scaled to the level of individual RMTs (Table 2), the mean TMS EF strength within the ROI was 75 ± 15 V/m, and the maximum EF strength was 207 ± 43 V/m.

**Figure 5:**
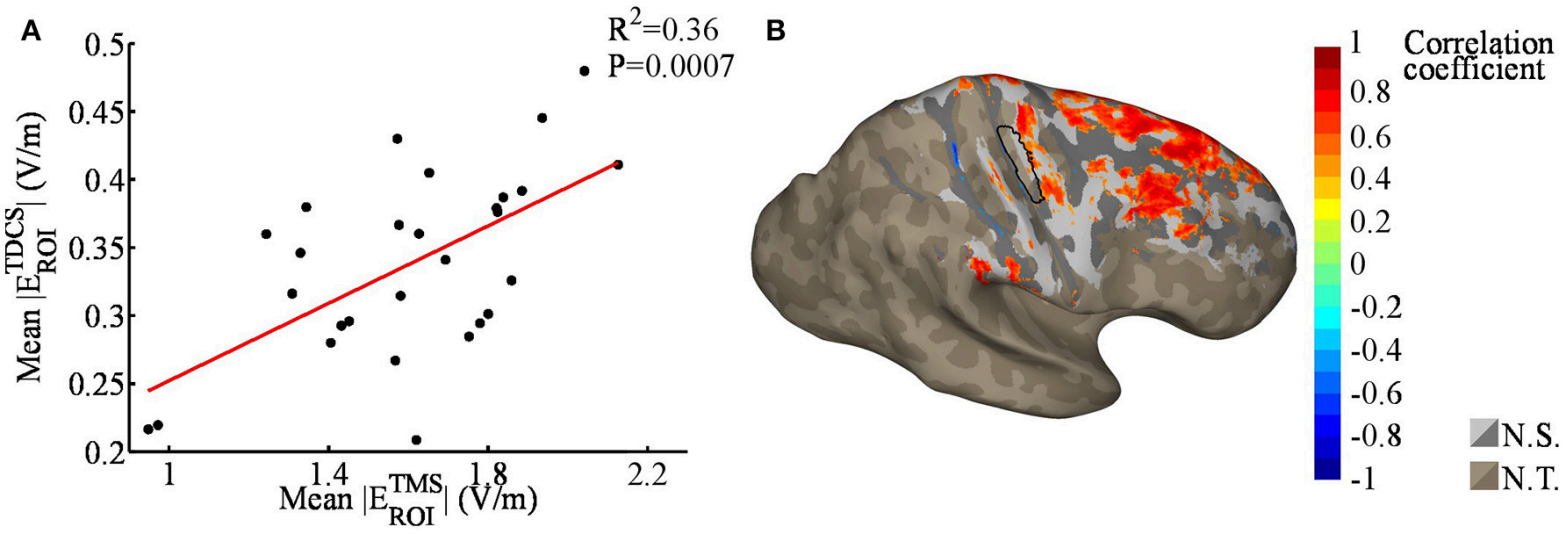
**(A)** Regression plot of the mean absolute transcranial direct current stimulation (TDCS) electric fields (EFs) within the region of interest [ROI; black outlined area in **(B)**], as a function of the mean absolute transcranial magnetic stimulation (TMS) EFs within the ROI. The TMS EFs were significantly correlated with the TDCS EFs. **(B)** Nodewise correlation between individual TDCS and TMS EFs; significant correlation coefficients are shown in color, the non-significant coefficients in gray (N.S.), and unstudied areas (average TDCS EF lower than 50% of the maximum) in brown (N.T.). The shading in the gray/brown areas represents the gyral structure of the brain (dark = sulci, light = gyri).

Linear regression analysis of the mean EF strengths in the ROI showed a significant correlation between the TMS and TDCS EFs (*R*^2^ = 0.36, *P* < 0.001, see Figure 5A). No outliers were detected. Our nodewise examination (see Figure 5B) of the correlation between the TDCS and TMS EFs revealed a significant (with a 5% FDR) positive correlation in a wide region of the cortex, mainly in the precentral gyrus and frontal areas. Spatially, there are significant correlations located also on the gyri anterior to the ROI. This is most likely due to these regions being far away from the sources of the EFs for both TMS and TDCS, and thus, the EFs in these regions might be similarly affected by the individual anatomy in both cases. Note that especially the TMS EFs are rather weak in the anterior regions (Figure 4B), where the highest EFs take place on the gyral crowns in similar manner to the TDCS EFs (Figure 2B).

**Figure 6:**
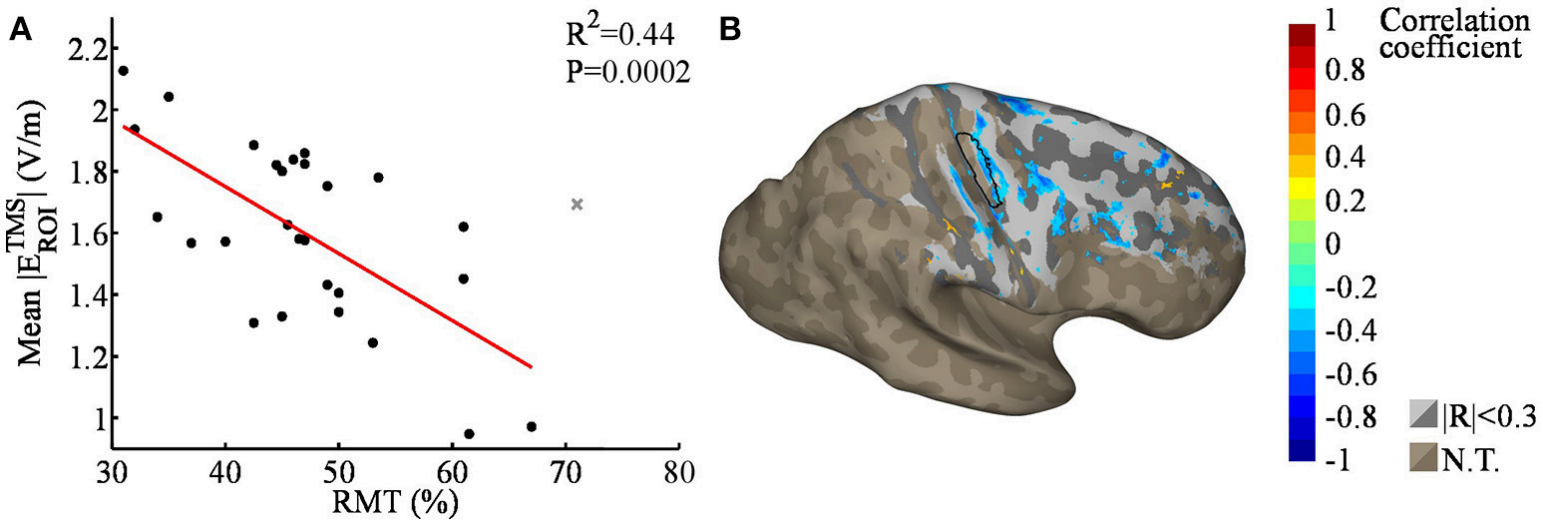
**(A)** Regression plot of the mean TMS EF strengths within the ROI [black outlined area in **(B)**], as a function of the RMT; the gray cross marks the outlier that was omitted from the analysis. The average TMS EF strength was found to correlate significantly with RMT. **(B)** Nodewise correlation between individual RMT and TMS EFs; correlation coefficients with |*R*| > 0.3 are shown in color, smaller coefficients in gray, and unstudied areas (average TDCS EF lower than 50% of the maximum) in brown (N.T.). The shading in the gray/brown areas represents the gyral structure of the brain (dark = sulci, light = gyri). None of the correlations were significant with 5% FDR.

We also studied the spatial correlation between the RMT and TMS EF strengths (Figure 6). Although the average TMS EF strength in the ROI was found to correlate with the RMT (*R*^2^ = 0.44, *P* < 0.001), no significant correlations were found in nodewise analysis using 5% FDR. Regions outside M1 did not seem to exhibit any systematic correlation between the TMS EF strength and RMT (Figure 6B), which is similar to the case with TDCS EFs (Figure 3B). This result is in line with the hypothesis that far from the sources, the EFs may be mainly affected by individual anatomic differences, not RMT.

## Discussion

In the present study, we measured the TMS RMTs and modeled realistic and individual TDCS and TMS EFs for 28 subjects. We found that the RMT was correlated with the modeled TDCS EF strength in hand M1 (*R*^2^ = 0.58, *P* < 0.001), a finding that has important implications for both the interpretation and design of TDCS experiments. No obvious correlations were identified in regions outside M1. The correlation between the calculated TDCS EF strengths and the measured RMTs that we identified beneath the TDCS anode may provide a simple method by which to estimate the TDCS EF strengths in hand M1 of individual subjects, making it a valuable tool for designing motor cortical TDCS protocols. Here, we found that the individuals with low RMTs tended to have larger TDCS-induced EFs in hand M1 than did subjects with high RMTs. This may be the physical mechanism underlying the recent findings of Jamil et al. (2017) and Labruna et al. (2016) showing that inter-individual sensitivity to TMS might affect the after-effects of anodal TDCS.

In order to understand the correlation between the TDCS EFs and RMT, we studied the relationship between the EFs of TDCS and TMS by modeling the TMS-induced EFs. A linear regression analysis revealed a positive correlation between the EF strengths of TMS and TDCS in hand M1. This positive correlation is interesting, because it suggests that both the stimulus (i.e., TDCS) and the method for measuring the effect of stimulation (i.e., TMS) are related to each other and that this relationship should be considered when designing TDCS experiments. Our results suggest that recipients of high TMS EFs may also receive high TDCS EFs, potentially biasing the experimental results of motor cortical TDCS toward subjects who are more sensitive to TMS. However, the TMS stimulator intensity in motor cortical experiments is typically either 110–150% of the RMT or set such that approximately 1 mV MEPs are produced at baseline (Horvath et al., 2015), thus approximately leveling the TMS EFs over subjects and minimizing the potential bias.

It should also be noted that the strength is not the only parameter when talking about the efficacy of TDCS in terms of EFs: also the polarity (anodal/cathodal) and the direction [parallel/perpendicular to the cortical surface (Rawji et al., 2018)] of the EFs should be taken into account when determining the dose. Thus further research is required in order to study whether the RMTs could also be used to predict the efficacy of TDCS. Also, only a single electrode montage for anodal/cathodal stimulation of the motor cortex was considered in this study, so care must be taken in extrapolating these results to other electrode montages and especially to other cortical target sites, as we found that RMT and TDCS EF strengths to correlate significantly only in the M1. Furthermore, instead of the usual approach used in measuring RMT, where the TMS coil is moved on the scalp to pinpoint the stimulation hot spot, we placed the coil guided solely by the MR images without attempting to find the hot spot.

Our computational modeling approach appears to be valid based on the relationship we identified between the measured data (RMTs) and the modeled EFs. However, there are a number of uncertainties inherent in the EF model, including the conductivity values (Akhtari et al., 2006, 2010; Laakso et al., 2016) and the segmentation process (Laakso et al., 2015). The amplitudes of the EFs we observed were within the same ranges as those found in previous simulation studies (Datta et al., 2009, 2012; Laakso et al., 2015, 2016), with the slight differences likely resulting from the different conductivity values and electrode sizes used in each study. In general, calculated EFs are higher than are those measured *in vivo* (Opitz et al., 2016; Huang et al., 2017), likely due to limitations in the conductivity values and to the experimental difficulties in measuring TDCS EFs *in vivo*.

## Conclusions

The present study revealed that TMS RMTs could be used as a simple measure for estimating the strength of TDCS EFs in hand M1. Subjects having higher RMTs tended to have lower TDCS EFs, implying that the RMT has the potential to serve as a meaningful tool for estimating the EF dose in motor cortical TDCS. Additionally, we demonstrated a correlation between the EFs of TDCS and TMS, suggesting that subjects who are more sensitive to TMS also have higher TDCS EFs in hand M1. Nevertheless, more research on how to convert the observed correlation between TDCS EF strengths and RMTs into a clinically useful method is required.

## Author contributions

IL, AH, and ST designed the study. SK and MS performed the measurements and analyzed the measured data. MM and IL performed the computational analysis and analyzed the data. MM, IL, and ST wrote the paper.

### Conflict of interest statement

The authors declare that the research was conducted in the absence of any commercial or financial relationships that could be construed as a potential conflict of interest.
